# Nuclear cGMP-Dependent Kinase Regulates Gene Expression via Activity-Dependent Recruitment of a Conserved Histone Deacetylase Complex

**DOI:** 10.1371/journal.pgen.1002065

**Published:** 2011-05-05

**Authors:** Yan Hao, Ningyi Xu, Andrew C. Box, Laura Schaefer, Kasthuri Kannan, Ying Zhang, Laurence Florens, Christopher Seidel, Michael P. Washburn, Winfried Wiegraebe, Ho Yi Mak

**Affiliations:** 1 Stowers Institute for Medical Research, Kansas City, Missouri, United States of America; 2 Department of Pathology and Laboratory Medicine, University of Kansas Medical Center, Kansas City, Kansas, United States of America; 3 Department of Molecular and Integrative Physiology, University of Kansas Medical Center, Kansas City, Kansas, United States of America; University of California San Diego, United States of America

## Abstract

Elevation of the second messenger cGMP by nitric oxide (NO) activates the cGMP-dependent protein kinase PKG, which is key in regulating cardiovascular, intestinal, and neuronal functions in mammals. The NO-cGMP-PKG signaling pathway is also a major therapeutic target for cardiovascular and male reproductive diseases. Despite widespread effects of PKG activation, few molecular targets of PKG are known. We study how EGL-4, the *Caenorhabditis elegans* PKG ortholog, modulates foraging behavior and egg-laying and seeks the downstream effectors of EGL-4 activity. Using a combination of unbiased forward genetic screen and proteomic analysis, we have identified a conserved SAEG-1/SAEG-2/HDA-2 histone deacetylase complex that is specifically recruited by activated nuclear EGL-4. Gene expression profiling by microarrays revealed >40 genes that are sensitive to EGL-4 activity in a SAEG-1–dependent manner. We present evidence that EGL-4 controls egg laying via one of these genes, Y45F10C.2, which encodes a novel protein that is expressed exclusively in the uterine epithelium. Our results indicate that, in addition to cytoplasmic functions, active EGL-4/PKG acts in the nucleus via a conserved Class I histone deacetylase complex to regulate gene expression pertinent to behavioral and physiological responses to cGMP. We also identify transcriptional targets of EGL-4 that carry out discrete components of the physiological response.

## Author Summary

Nitrates and phosphodiesterase inhibitors raise the intracellular level of cGMP, and they have been widely used to treat hypertension and erectile dysfunction. Although it is known that cGMP activates the cGMP-dependent protein kinase PKG, which in turn causes smooth muscle relaxation and other physiological responses, very few molecular targets of PKG have been identified. In addition, the long-term effects of sustained elevation of cGMP and PKG activation are not known. We study a family member of PKG called EGL-4 in the nematode *C. elegans*. Using a combination of unbiased forward genetic screen and proteomic analysis, we show that constitutively active EGL-4 alters gene expression in multiple tissues, which is achieved through activity-dependent recruitment of a conserved Class I histone deacetylase complex in the nucleus. Furthermore, we identify a novel EGL-4–responsive gene that encodes a putative secreted protein that modulates the egg laying rate of *C. elegans*. Taken together, our results uncover novel PKG targets in the nucleus that respond to sustained elevation of cGMP. Development of chemicals that modulate the activity of these PKG targets may differentiate or alleviate undesirable side-effects of existing drugs that manipulate cGMP level.

## Introduction

The cellular level of cGMP is controlled by a balance of guanylyl cyclase and phosphodiesterase activities [1], [2]. For example, nitric oxide (NO) activates soluble guanylyl cyclases to generate cGMP, which ultimately causes vasodilation and lowering of blood pressure. The cGMP dependent protein kinase PKG is one of the key effectors of cGMP signaling [3]. Although the physiological roles of mammalian PKGs had been extensively studied using genetically engineered mice, few molecular targets of PKGs were discovered to date that could account for the extensive effect of PKG activation in cardiovascular, digestive and nervous systems [3]. We study the *C. elegans* PKG ortholog, EGL-4, in order to discover additional evolutionarily conserved molecular effectors of cGMP-PKG singaling.

Environmental conditions dictate larval developmental decisions and a number of adult behaviors such as foraging and egg laying in the free living nematode *C. elegans*. Animals navigate towards food source and away from unfavorable growth conditions, such as sub-optimal temperature, high population density and pathogenic organisms [4]–[6]. This is achieved through detection of environmental conditions by dedicated sensory neurons and integration of signals in higher order neurons, which then instruct physiological and locomotory responses via efferent neurons and neuroendocrine signals. In *C. elegans*, insulin, TGF-β and cGMP signaling pathways have been implicated in organismal homeostasis in response to changes in environmental conditions [7]–[12]. While the effectors of insulin and TGF-β pathways have been elucidated by genetic and biochemical analysis [13]–[16], how cGMP signaling leads to coordinated physiological responses throughout the body is poorly understood. It is known that cGMP activates cyclic nucleotide gated channels (CNGs) that alter membrane potential of neurons [17]–[19]. In addition, cGMP activates cGMP dependent protein kinase (PKG), whose downstream effectors that mediate coordinated physiological responses to environmental and developmental signals have not been identified in *C. elegans*.

Mutant alleles of the *C. elegans* cGMP dependent protein kinase EGL-4 were originally identified from a genetic screen for egg laying defective mutants [20]. Subsequently, EGL-4 was found to regulate diverse processes such as chemotaxis, olfactory adaptation, foraging behavior, body length, dauer arrest and adult life-span [21]–[25]. It has also been reported that EGL-4 is required for behavioral quiescence, in response to food or ectopic epidermal growth factor signaling [12], [26], [27]. Regulation of foraging behavior by PKG appears to be conserved in *Drosophila* as the expression level of the PKG ortholog *foraging* dictates sitter versus rover phenotype in larvae [28]. While EGL-4 acts in sensory neurons to modulate foraging behavior [22], [24], EGL-4 is also expressed throughout the body and regulates body length from a number of tissues [29]. Major questions remain regarding how EGL-4 activity is transduced in different tissues throughout the body. For example, the time-scale of various physiological responses following EGL-4 activation is unknown. In addition, the cytoplasmic versus nuclear function of EGL-4, and its molecular effectors in these subcellular compartments have not been clearly defined.

The *C. elegans* EGL-4 is most homologous to mammalian PKG-Iβ, which has an auto-inhibitory domain at its N-terminus, followed by two cGMP binding domains and a kinase domain [3]. PKG is normally held inactive by its auto-inhibitory domain and is activated through cooperative binding of cGMP, whose level is controlled by opposing action of guanylyl cyclase and phosphodiesterase [1], [2]. PKG is known to regulate smooth muscle tone in response to elevation of cGMP as a result of soluble guanylyl cyclase activation by nitric oxide [3]. A handful of PKG targets have been identified that account for the cytoplasmic role of PKG in smooth muscles [3], [30]–[32]. In addition, it has been suggested that PKG activity may also affect gene expression in cell culture systems [33], [34]. However, the molecular mechanisms that underlie nuclear PKG activity are not fully understood.

To address how EGL-4 and its downstream effectors may coordinate physiological responses upon changes in environmental conditions, we characterized a *C. elegans* mutant that expresses a constitutively active EGL-4/PKG. Our analyses revealed genomic effects of EGL-4 activity via a conserved histone deacetylase complex. We also present evidence that EGL-4 activity can modulate the expression of a novel extracellular signaling protein, which illustrates how EGL-4 may exert cell non-autonomous effects.

## Results

### EGL-4/PKG regulates foraging behavior and egg-laying rate

**Figure 1:**
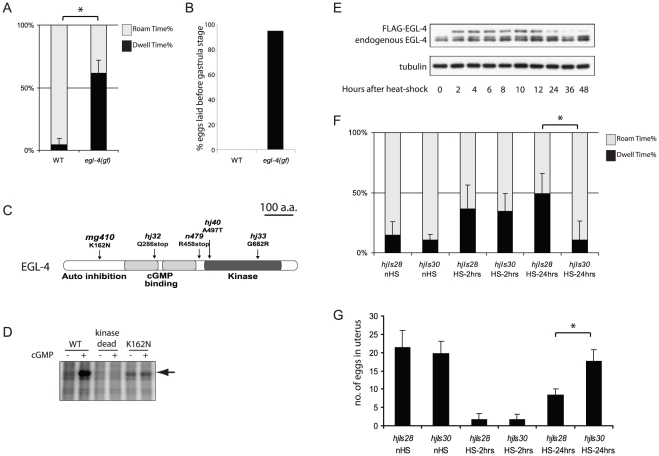
EGL-4/PKG controls foraging behavior and egg laying. (A) Foraging behavior of wild-type and *egl-4(gf)* mutant animals were monitored in 15-minute intervals. The percentage time roaming or dwelling of each animal was quantitated using the Worm Tracker program. 5 animals were monitored in each trial. Total number of trials: n = 25 (wild type); n = 19 (*egl-4(gf)*). (Mean±SD; pair-wise t-test, *, *p<0.05*). (B) Percentage of eggs laid prior to gastrula stage within a 1-hr period. Total number of adult animals (total number of eggs observed): wild type =  10(108); *egl-4(mg410)* =  20(77). (C) Schematic representation of EGL-4/PKG. Mutant alleles together with corresponding changes in the protein coding sequence in the EGL-4A isoform are indicated. (D) Autoradiograph from an *in vitro* kinase assay with wild-type (WT) and mutant forms of EGL-4 that were expressed in *Drosophila* S2 cells. Arrow indicates auto-phosphorylated EGL-4 that is absent in the kinase dead control. (E) Expression level of EGL-4 in lysates prepared from transgenic animals carrying *hjIs28[hsp::3xFLAG::EGL-4(K162N)::SL2::mCherry]* at specified time after heat shock at 33°C for 30 mins. Endogenous and FLAG-tagged EGL-4 protein was detected using anti-EGL-4 antibody. The α-tubulin blot served as loading control. (F) Foraging behavior of wild-type animals carrying the transgene *hjIs28[hsp::3xFLAG::EGL-4(K162N)::SL2::mCherry]* that expressed constitutively active EGL-4 or *hjIs30[hsp::3xFLAG::EGL-4(K499A)::SL2::mCherry]* that expressed kinase-dead EGL-4. Quantitation of behavior was performed as in (A). Total number of trials: n = 6 (non-heatshock (nHS) control and 2 hrs after heatshock (HS-2hrs)); n = 13 (24 hrs after heatshock (HS-24hrs)). (Mean±SD; pair-wise t-test, *, *p<0.05*). (G) Number of eggs retained in uterus in transgenic animals carrying *hjIs28* or *hjIs30*. 24 hrs after heatshock, significantly fewer eggs were retained in animals in which constitutively active EGL-4 had been ectopically expressed (*hjIs28*), indicating a faster egg-laying rate. Total number of animals for each strain at each time point: n = 20. (Mean±SD; pair-wise t-test, *, *p<0.05*)

We isolated *mg410*, a dominant *egl-4* gain-of-function allele from a genetic screen for mutants with elevated Nile Red staining in the absence of a functional peroxisomal thiolase, *kat-1* [35]. The *egl-4(mg410)* mutant animals display pleiotropic phenotypes such as excessive dwelling and reduced body length (Figure 1A, Table 1). These animals also have elevated egg-laying rate, since they lay eggs with embryos younger than the gastrula stage and as early as the 8-cell stage (Figure 1B) and the number of eggs in their uterus is significantly reduced (Table 1). Molecular cloning revealed that *mg410* encoded a single amino acid substitution K162N (Figure 1C). Lysine 162 in EGL-4 is a key residue of a conserved pseudo-substrate motif that was shown to be critical for cGMP dependent activation of mammalian PKG [36]. Mutation of the pseudo-substrate motif causes constitutive activation of PKG and auto-phosphorylation [36]. Accordingly, EGL-4(K162N) underwent auto-phosphorylation in the presence or absence of cGMP in an *in vitro* kinase assay (Figure 1D). Auto-phosphorylation was not detected in the EGL-4(K499A) kinase dead mutant control.

**Table 1:** Body length and number of eggs in uterus in wild-type and mutant animals.

Genotype	Body length (µm)		SD	n	no. of eggs in uterus		SD	n
*N2*	1108.79	±	54.54	160	18.20	±	5.36	82
*egl-4(n479)*	1294.81	±	35.55	23	47.36	±	9.87	39
*egl-4(mg410)*	685.33	±	31.03	101	3.15	±	1.51	40
*egl-4(mg410hj32)*	1342.17	±	35.76	20	56.48	±	11.20	29
*egl-4(mg410hj33)*	1050.75	±	40.00	17	13.59	±	3.11	29
*egl-4(mg410hj40)*	1125.69	±	64.39	19	18.00	±	5.31	40
*saeg-1(hj12)*	1028.27	±	38.42	20	10.40	±	3.44	20
*egl-4(mg410); saeg-1(hj12)*	1026.49	±	53.93	20	9.88	±	3.63	40
*saeg-2(hj9)*	1044.11	±	44.98	20	12.00	±	3.40	19
*egl-4(mg410); saeg-2(hj9)*	931.46	±	31.71	20	11.48	±	3.30	40
*saeg-2(ok3174)*	1071.22	±	38.53	37	6.65	±	2.16	20
*egl-4(mg410); saeg-2(ok3174)*	964.71	±	41.03	40	9.20	±	1.70	20
*egl-4(mg410); saeg-1(hj12); saeg-2(ok3174)*	1036.25	±	33.17	20	13.68	±	4.02	22
*egl-4(mg410); saeg-2(ok3174); hjSi15*	771.08	±	28.46	20	4.90	±	1.29	20
*hda-2(ok1479)*	1034.14	±	96.40	20	17.05	±	6.02	20
*egl-4(mg410); hda-2(ok1479)*	801.54	±	41.60	40	5.55	±	2.34	40
*egl-4(mg410); hda-2(ok1479); Ex[hda-2(+)]a*	693.66	±	46.12	20	3.53	±	1.57	38
*egl-4(mg410); hda-2(ok1479); Ex[hda-2(+)]b*	700.86	±	39.59	19	4.72	±	2.53	25
*egl-4(mg410); hda-3(ok1991)*	681.14	±	37.12	20	2.78	±	0.77	40

To confirm that the *mg410* allele, and the corresponding K162N substitution, is responsible for the pleiotropic phenotypes associated with constitutive activation of EGL-4, we isolated multiple intragenic *egl-4(mg410)* suppressor alleles. Among them, *hj32*, a null allele that encodes a pre-mature stop codon for a complete loss of EGL-4 protein (Figure 1C, Figure S1A). The *hj32* allele confers strong *egl-4* loss-of-function phenotypes similar to the canonical allele *n479* [22] (Table 1). In addition, we identified two weak *egl-4* loss-of-function alleles, *hj33* and *hj40*. These are missense alleles that encode single amino acid substitutions in the EGL-4 kinase domain (Figure 1C). The *egl-4(mg410hj33)* and *egl-4(mg410hj40)* mutant animals are phenotypically similar to wild-type animals (Table 1), suggesting that these missense alleles confer a partial loss of EGL-4 kinase activity.

Foraging behavior and egg laying rate are controlled by dedicated neuronal circuits that couple sensory inputs with motor outputs. It is plausible that the pleiotropic phenotypes of the *egl-4* gain-of-function *(gf)* mutant are due to developmental defects of specific neuronal circuits. Alternatively, EGL-4 may regulate foraging behavior and egg laying frequency by affecting the activity of pre-established circuits. To distinguish between these possibilities, we transiently expressed a constitutively active (K162N) or kinase dead (K499A) form of EGL-4 that carries a FLAG-epitope tag under the control of a heat-shock promoter in wild-type animals. These animals were allowed to develop to young adult stage when all neuronal circuits are established. After heat-shock, the FLAG-tagged EGL-4 reached steady-state level in 2 hours and started waning after 12 hours (Figure 1E, Figure S1B and S1C). Expression level of the FLAG-tagged EGL-4 was comparable to the endogenous protein (Figure 1E). Using the Worm Tracker program for quantitative analysis of foraging behavior [37], we found no significant difference in foraging behavior between wild-type animals and animals that expressed constitutively active (*hjIs28*) or kinase dead EGL-4 (*hjIs30*), 2 hours after heat-shock when the expression level of ectopic EGL-4 peaked (Figure 1F, Figure S1D). The percentage dwell time of all three strains increased, which might be caused by the heat-shock treatment 2 hours prior to our measurement. However, ectopic expression of the constitutively active EGL-4 (*hjIs28*) caused excessive dwelling when compared with wild-type animals and the kinase dead control (*hjIs30*) 24 hours after heat-shock (Figure 1F, Figure S1D). The excessive dwelling was comparable to that of *egl-4(gf)* mutant animals. The same temporal effect was observed on egg laying frequency upon ectopic expression of a constitutively active EGL-4 (Figure 1G, Figure S1E). We concluded that excessive dwelling and elevated egg laying frequency in *egl-4(gf)* animals were not due to a defect in neuronal circuit assembly. Given the delayed effects of EGL-4 activation, we hypothesize that EGL-4 may mount a transcriptional program that triggers sustained changes on established neuronal circuits. Nevertheless, we cannot rule out a role for EGL-4 in regulating short-term synaptic activity.

### SAEG-1 and SAEG-2 act downstream of EGL-4/PKG

**Figure 2:**
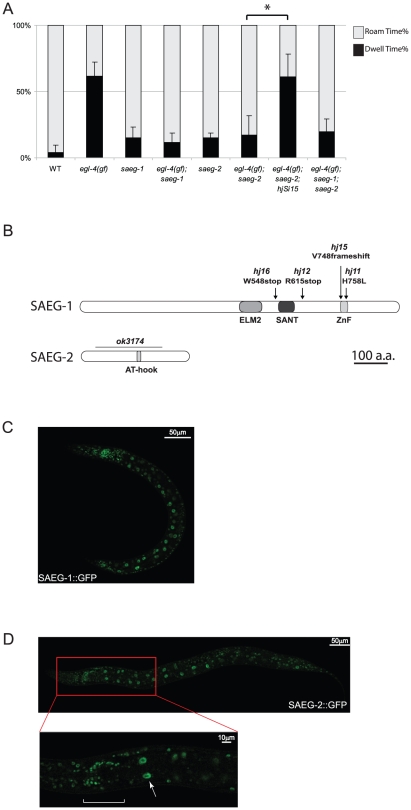
SAEG-1 and SAEG-2 act downstream of EGL-4 to control foraging behavior. (A) Foraging behavior of wild-type (WT), *egl-4*, *saeg-1* and *saeg-2* mutant animals. Quantitation of behavior was performed as in Figure 1A. Data for WT and *egl-4(gf)* animals are the same as in Figure 1A. Total number of trials for all other strains: n = 5. (Mean±SD; pair-wise t-test, *, *p<0.05*). (B) Schematic representations of SAEG-1 and SAEG-2 protein. Conserved domains are indicated; ZnF, C2H2 zinc finger. Mutant alleles together with corresponding changes in protein coding sequences are indicated. The *saeg-2(hj9)* molecular lesion is not shown because it affects the splice donor site of intron 3. (C) Nuclear localization of SAEG-1::GFP fusion protein. Confocal image of a larval L4 stage *egl-4(gf); saeg-1; Ex[saeg-1p::saeg-1::gfp]* animal is shown. (D) Nuclear localization of SAEG-2::GFP fusion protein. Confocal image of a larval L4 stage *saeg-2; hjSi15[saeg-2p::saeg-2::gfp]* animal is shown. Inset shows the same animal at higher magnification, bracket indicates nuclei of neurons in the nerve ring and arrow indicates an intestinal nucleus.

To identify downstream effectors of the activated EGL-4 kinase, we mutagenized *egl-4(gf)* animals for mutant alleles that suppressed their excessive dwelling behavior. These alleles fell into two complementation groups, defining two suppressor of activated EGL-4 (*saeg-*) genes. Several lines of evidence suggest that *saeg-1* and *saeg-2* are likely to encode ubiquitous downstream effectors of EGL-4. First, recessive mutations in *saeg-1* and *saeg-2* genes strongly suppressed excessive dwelling, accelerated egg laying rate and short body length of *egl-4(gf)* animals (Figure 2A, Table 1). Second, loss of *saeg-2* function suppressed the behavioral phenotypes triggered by ectopic expression of a constitutively active EGL-4 (Figure S1D and S1E). Third, loss of *saeg-1* or *saeg-2* function did not drastically alter *egl-4* mRNA levels and more importantly, endogenous EGL-4 protein level remained constant (Figure S2A and S2B). Since there is no phenotypic difference between *egl-4(gf)*; *saeg-1*, *egl-4(gf)*; *saeg-2* and *egl-4(gf)*; *saeg-1*; *saeg-2* mutant animals (Figure 2A, Table 1), we conclude that *saeg-1* and *saeg-2* act in the same genetic pathway.

We isolated four recessive alleles of *saeg-1*. Molecular cloning revealed that *saeg-1* encodes a C2H2 zinc-finger protein that also contains the conserved ELM2 [38] and SANT domains [39] (Figure 2B). Based on sequence homology and molecular architecture, SAEG-1 is orthologous to human transcriptional regulating factor 1 (TRERF1; Gene ID: 55809) and zinc-finger protein 541 (ZNF541; Gene ID: 84215) [40], [41]. We generated a rescuing transgene using 29 kb of genomic sequence that encompassed the *saeg-1* gene and inserted the green fluorescent protein (GFP) coding sequence immediately 5′ to the *saeg-1* stop codon. We found that SAEG-1::GFP is a ubiquitous nuclear protein (Figure 2C). The zinc-finger is required for SAEG-1 function because animals carrying the *hj11* allele, which encodes a leucine substitution for one of the zinc coordinating histidines, is phenotypically indistinguishable to those carrying the *hj12* allele, a nonsense allele that causes severe reduction of *saeg-1* transcript level. We focused our phenotypic analysis on *saeg-1(hj12)* animals.

The *saeg-2* gene was defined by a single recessive allele, *hj9*, from our genetic screen. The *hj9* allele encodes a guanine to adenine substitution that eliminates the consensus splice donor site of the third intron. A second allele of *saeg-2*, *ok3174*, was isolated by the *C. elegans* Knockout Consortium. Both *hj9* and *ok3174* alleles are molecular null alleles because no SAEG-2 protein is detectable in animals carrying either alleles, which show comparable phenotypes (Table 1, Figure S2C). The SAEG-2 protein is orthologous to mammalian terminal deoxynucleotidyltransferase interacting protein 1 (Dnttip1; Gene ID: 116092) [42]. We generated a rescuing, single-copy transgene *hjSi15* that expressed a SAEG-2::GFP fusion protein under the control of the endogenous *saeg-2* promoter. Similar to SAEG-1::GFP, SAEG-2::GFP is expressed ubiquitously and is localized to the nucleus (Figure 2D). The nuclear localization of SAEG-2::GFP is not dependent on EGL-4 activity (Figure S2D). Taken together, our results suggest that SAEG-1 and SAEG-2 are nuclear effectors of EGL-4, perhaps by modulating gene expression in response to EGL-4 activity. This is consistent with the latent manifestation of foraging and egg laying phenotypes upon ectopic expression of an activated form of EGL-4.

### Physical interaction between EGL-4/PKG, SAEG-1, and SAEG-2 is conserved

**Figure 3:**
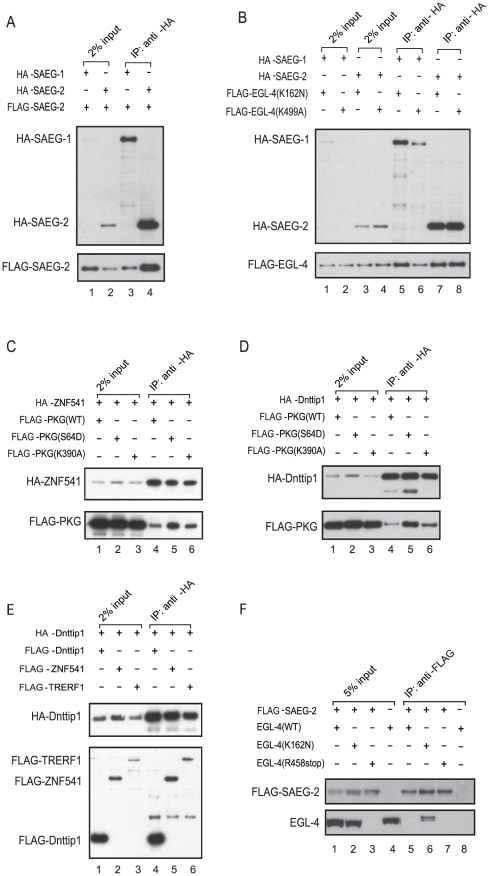
Physical interaction between EGL-4, SAEG-1, SAEG-2, and their mammalian orthologs. (A) Co-immunoprecipitation of FLAG-tagged SAEG-2 (FLAG-SAEG-2) with HA-tagged SAEG-1 (HA-SAEG-1) or SAEG-2 (HA-SAEG-2) upon co-expression in *Drosophila* S2 cells. (B) Co-immunoprecipitation of FLAG-tagged EGL-4 with HA-tagged SAEG-1 (HA-SAEG-1) or SAEG-2 (HA-SAEG-2) upon co-expression in *Drosophila* S2 cells. SAEG-1 and SAEG-2 associate with constitutively active (K162N) or kinase dead (K499A) EGL-4 when over-expressed. (C) Co-immunoprecipitation of FLAG-tagged wild-type (WT), constitutively active (S64D) or kinase dead (K390A) PKG-Iβ with HA-tagged ZNF541 after co-expression in HEK293 cells. (D) Co-immunoprecipitation of FLAG-tagged wild-type (WT), constitutively active (S64D) or kinase dead (K390A) PKG-Iβ with HA-tagged Dnttip1 after co-expression in HEK293 cells. (E) Co-immunoprecipitation of FLAG-tagged Dnttip1, ZNF541 or TRERF1 with HA-tagged Dnttip1 after co-expression in HEK293 cells. (F) Preferential association of endogenous, constitutively active EGL-4(K162N) with FLAG-tagged SAEG-2 that was expressed at the endogenous level. Mutant animals carrying the *egl-4(n479)* allele did not express full length EGL-4 protein. Five independent experiments were performed and results from one representative experiment are shown.

We observed that endogenous, wild-type or constitutively active EGL-4 kinase is enriched in the nucleus (Figure S3). Nuclear EGL-4::GFP fusion protein has also been observed in most neurons [43]. Given that SAEG-1 and SAEG-2 are nuclear localized (Figure 2C and 2D), we wondered if EGL-4/PKG, SAEG-1 and SAEG-2 could physically interact with each other. Indeed, the mammalian orthologs of SAEG-1 and SAEG-2, TRERF1 and Dnttip1 respectively, have been shown to interact with each other *in vitro* [44]. We confirmed that SAEG-2 co-immunoprecipitated with itself and with SAEG-1, but not the yellow fluorescent protein Venus when over-expressed in *Drosophila* S2 cells (Figure 3A, lanes 3 and 4; Figure S4A, lanes 2 and 4). Furthermore, SAEG-1 and SAEG-2 specifically co-immunoprecipitated with constitutively active or kinase dead forms of EGL-4 in the same over-expression system (Figure 3B, lanes 5-8, Figure S4B, lanes 8, 10 and 12). We also found that the interactions between EGL-4, SAEG-1 and SAEG-2 are conserved for their mammalian orthologs (Figure 3C–3E, Figure S4C and S4D). Finally, we tested the interaction between endogenous SAEG-2 and EGL-4 when both were expressed at physiological level in *C. elegans*. We found that endogenous, activated EGL-4 from *egl-4(gf)* animals specifically associated with SAEG-2 (Figure 3F, lane 6). In contrast, there was no physical association between SAEG-2 and EGL-4 in its basal state as found in wild-type animals under the same experimental conditions (Figure 3F, lane 5). Our results indicate that EGL-4 modulates gene expression by recruiting the SAEG-1/SAEG-2 complex in an activity dependent manner.

### SAEG-1 and SAEG-2 form a conserved histone deacetylase complex

**Figure 4:**
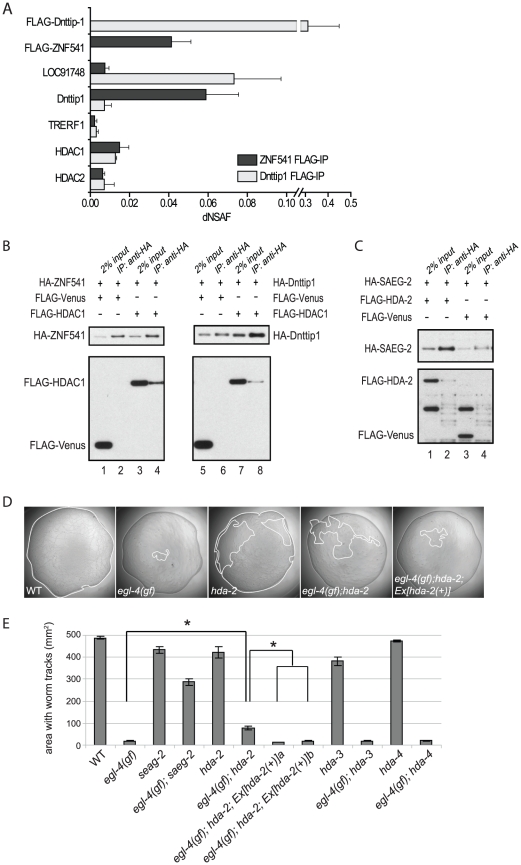
HDA-2 is part of the SAEG-1/SAEG-2 complex that mediates EGL-4 activity. (A) MudPIT analysis on proteins co-immunoprecipitated with FLAG-tagged Dnttip1 or ZNF541 from HEK293 cells. dNASF (distributed normalized spectral abundance factors) indicates the relative abundance of proteins. Results are from 3 independent biological samples. (Mean±SD). (B) Co-immunoprecipitation of FLAG-tagged HDAC1 with HA-tagged ZNF541 or Dnttip1 after co-expression in HEK293 cells. (C) Co-immunoprecipitation of FLAG-tagged HDA-2 with HA-tagged SAEG-2 after co-expression in *Drosophila* S2 cells. (D) Representative images of bacterial lawns on which a single animal was placed for 18 hours at 20°C. Areas covered by worm tracks (enclosed by a white line) were measured as an indicator of foraging behavior of individual animals. Excessive dwelling behavior by *egl-4(gf)* animals resulted in the smallest area in which worm tracks were found. (E) Quantitation of bacterial lawn area that was explored by individual animals of indicated genotypes. Number of animals tested: n = 60 (wild type and *egl-4(gf)*); n = 30 (all other genotypes). (Mean±SD; pair-wise t-test, *, *p<0.05*)

Since the interaction between SAEG-1 and SAEG-2 is conserved from *C. elegans* to mammals, we asked whether SAEG-1 and SAEG-2 and their mammalian orthologs are part of a larger protein complex that mediates EGL-4/PKG activity. We established human embryonic kidney 293 cell lines that stably expressed either FLAG-epitope tagged ZNF541 (SAEG-1 ortholog) or Dnttip1 (SAEG-2 ortholog). Immunoprecipitation was followed by Multi-Dimensional Protein Identification Technology (MuDPIT) analysis [45], in order to identify proteins that associated with ZNF541 and Dnttip1 in independent samples (Figure 4A). In agreement with our previous results, endogenous Dnttip1 was detected in the ZNF541 protein complex, while endogenous TRERF1 and LOC91748, another protein containing ELM2-SANT domains, were found in the Dnttip1 protein complex. In addition, we found that histone deacetylase 1 (HDAC1) and histone deacetylase 2 (HDAC2) were the only proteins that consistently associated with both ZNF541 and Dnttip1. We verified the interaction between HDAC1, ZNF541 and Dnttip1 (Figure 4B, lanes 4 and 8) and concluded that ZNF541 and Dnttip1 are components of a novel histone deacetylase complex. The *C. elegans* HDA-2 is the closest ortholog of mammalian HDAC1 and HDAC2 and it co-immunoprecipitated with SAEG-2 when over-expressed in *Drosophila* S2 cells (Figure 4C, lane 2). However, we did not detect significant association between SAEG-1 and HDA-2 (YAH and HYM, unpublished data). It is plausible that SAEG-2 serves as an anchor by simultaneously interacting with SAEG-1 and HDA-2.

If the association of HDA-2 with SAEG-1 and SAEG-2 is required to mediate EGL-4 activity, then a loss of *hda-2* function should mimic the loss of *saeg-1* or *saeg-2*. Indeed, loss of *hda-2* function partially suppressed the excessive dwelling, accelerated egg laying and short body length phenotypes of *egl-4(gf)* animals (Figure 4D and 4E, Table 1). Suppression of *egl-4(gf)* phenotypes by loss of *hda-2* function could be reversed with an *hda-2p::hda-2(+)* transgene, demonstrating the specificity of such genetic interaction (Figure 4D and 4E, Table 1). Furthermore, loss of *hda-3* or *hda-4*, which encodes two closely related histone deacetylases, failed to suppress the *egl-4(gf)* phenotypes (Figure 4E, Table 1). Taken together, our results suggest that SAEG-1, SAEG-2 and HDA-2 form a histone deacetylase complex that is a nuclear effector of EGL-4 activity.

### Regulation of egg-laying rate by an EGL-4–responsive gene

**Figure 5:**
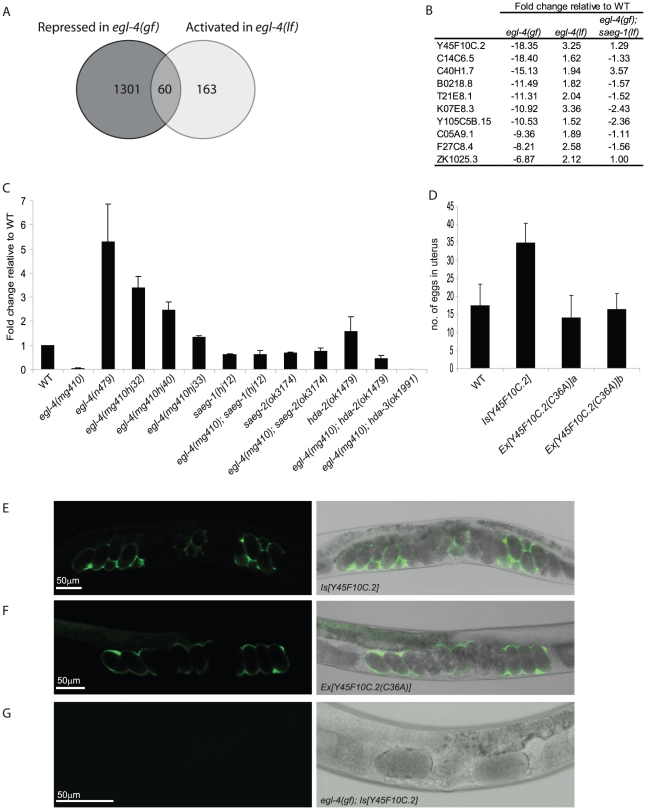
Y45F10C.2 is an EGL-4 activity responsive gene that regulates egg laying. (A) Gene expression profiling using Affymetrix microarray discovered 60 genes that were repressed in *egl-4(gf)* and activated in *egl-4(lf)* animals when compared with wild-type animals, using a cut-off of 1.5-fold change, *p<0.05*. (B) Top ten differentially expressed genes in *egl-4(gf)* versus *egl-4(lf)* animals. Fold change based on microarray analysis of three independent samples of each genotype is shown. (C) Real-time PCR analysis of Y45F10C.2 gene expression in wild-type and *egl-4* mutant animals at young adult stage. At least 2 independent RNA samples were analyzed for each genotype and the expression level in wild-type animals (WT) was set as 1. (D) Number of eggs retained in uterus in transgenic animals over-expressing wild-type or mutant Y45F10C.2 transgenes. Number of animals tested: n = 19 (WT and *Ex[Y45F10C.2(C36A)]* line A); n = 18 (*Is[Y45F10C.2]* and *Ex[Y45F10C.2(C36A)]* line B). (E) Representative image of a 2-day old adult animal carrying a *Y45F10C.2p::Y45F10C.2::SL2::GFP-PEST* transgene. Overexpression of Y45F10C.2 caused retention of eggs in the uterus. (F) Representative image of a 2-day old adult animal carrying a *Y45F10C.2p::Y45F10C.2(Cys36Ala)::SL2::GFP* transgene. Overexpression of mutant Y45F10C.2 did not affect egg-laying. (G) Representative image of a 2-day old adult *egl-4(gf)* animal carrying a *Y45F10C.2p::Y45F10C.2::SL2::GFP-PEST* transgene. Expression of the transgene was repressed.

Our results point to a molecular pathway in which activated EGL-4/PKG kinase engages a conserved histone deacetylase complex that modulates expression of key regulators of foraging behavior and egg laying rate. We hypothesized that such EGL-4 activity responsive genes are most likely repressed in *egl-4(gf)* animals in a SAEG-1/SAEG-2 dependent manner. These genes should also be de-repressed or activated in *egl-4(lf)* animals. Therefore, we performed gene expression profiling using Affymetrix microarrays on RNA samples extracted from wild-type, *egl-4(gf)*, *egl-4(lf)* and *egl-4(gf)*; *saeg-1(lf)* mutant animals at the late larval L4 and young adult stage. By focusing on genes that showed >1.5-fold change in expression (*p<0.05*) when compared with wild-type samples, we identified 60 genes that were repressed in *egl-4(gf)* and activated in *egl-4(lf)* animals (Figure 5A). The expression of 43 out of these 60 genes was normalized close to wild-type level (<1.5-fold change) in *egl-4(gf)*; *saeg-1(lf)* animals. In addition, 5 out of these 60 genes were de-repressed and activated (>1.5-fold) in the same animals. These results demonstrate that EGL-4 modulates gene expression almost entirely through SAEG-1, and by inference the SAEG-1/SAEG-2 complex. Although we have identified a group of genes whose expression was highly responsive to EGL-4 activity, our results did not imply direct recruitment of the SAEG-1/SAEG-2 complex to their promoters.

We focused our analysis on Y45F10C.2 as an EGL-4 activity responsive gene because it showed the maximal differential expression in *egl-4(gf)* versus *egl-4(lf)* animals (Figure 5B). Furthermore, Y45F10C.2 expression is de-repressed in *egl-4(gf)*; *saeg-1(lf)* animals. We verified the gene expression changes in Y45F10C.2 by quantitative real-time PCR (Figure 5C). The Y45F10C.2 gene encodes a novel protein with a predicted secretory peptide and it belongs to the DUF1505 family whose members contain 6 highly conserved cysteine residues. A duplicate of Y45F10C.2, C08F11.12, was found 10 kb away from its 3′ end. The Y45F10C.2 and C08F11.12 genes differ by a single nucleotide polymorphism in the coding sequence and their 5′ intergenic regulatory sequences are >98% identical over ∼775 bp. Our microarray and real-time PCR analysis allowed us to monitor the expression changes in both genes simultaneously and we found that the two genes had similar function (NXU and HYM, unpublished data). For simplicity, we will use Y45F10C.2 as a representative gene name hereafter.

The expression level of Y45F10C.2 closely correlated with the egg laying phenotype of our allelic series of *egl-4* mutant animals and by extension the EGL-4 activity (Figure 5C). Mild elevation of egg-laying rate in *saeg-1* or *saeg-2* single mutant animals also correlated well with a ∼2-fold reduction of Y45F10C.2 expression level (Figure 5C). Using a bi-cistronic transgene in which Y45F10C.2 and GFP expression were under the control of the Y45F10C.2 promoter, we found that the promoter was most active in the uterine epithelium (Figure 5E). It is plausible that EGL-4 regulates Y45F10C.2 in a cell-autonomous manner, since endogenous nuclear EGL-4 was detected in uterine epithelial cells (Figure S5).

Over-expression of Y45F10C.2 also caused a severe reduction in egg-laying rate and retention of fertilized eggs, a phenotype shared by *egl-4(lf)* animals where Y45F10C.2 expression was high (Figure 5D and 5E). The egg-laying phenotype triggered by Y45F10C.2 over-expression was specific because substitution of a highly conserved cysteine with alanine (Cys36Ala) in Y45F10C.2 abolished its ability to promote egg retention (Figure 5D and 5F). We note that Y45F10C.2 may be sufficient but not necessary for modulating egg-laying rate, because knock-down by RNA interference (RNAi) did not affect egg-laying in wild-type animals, perhaps due to functional compensation by other DUF1505 family members (NXU and HYM, unpublished data). Finally, the Y45F10C.2 transgene was not expressed in *egl-4(gf)* animals, consistent with the observation that the endogenous Y45F10C.2 gene was highly repressed in these animals (Figure 5G). Our results suggest that Y45F10C.2 is a target gene that regulates egg laying in response to EGL-4 activity and it may act in parallel of other EGL-4 effectors and the neuronal circuit that controls vulval muscles contraction.

## Discussion

**Figure 6:**
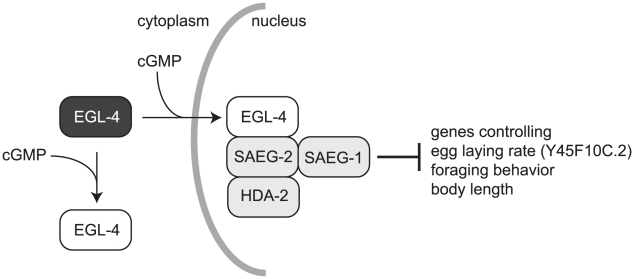
Genomic effects of activated nuclear EGL-4 are mediated by the SAEG-1/SAEG-2/HDA-2 complex.

In this paper, we combine genetic, proteomic and genomic approaches to determine how a cGMP dependent protein kinase, EGL-4, modulates gene expression. Activated EGL-4 preferentially associates with a conserved SAEG-1/SAEG-2 histone deacetylase complex, which in turn represses a novel gene that regulates egg-laying rate (Figure 6). Our results demonstrate that in additional to cytoplasmic roles in *Drosophila* and mammals [3], [46], cGMP dependent kinase can act in the nucleus to elicit long-term transcriptional changes that affect multiple physiological processes such as foraging behavior, egg laying rate and body length in *C. elegans*.

We propose that EGL-4 plays a central role in linking sensory input regarding environmental conditions to long-term physiological responses by altering gene expression of intracellular and extracellular signaling molecules. Although nuclear function of EGL-4 and mammalian PKG had been suggested in the past, in part through the demonstration of EGL-4/PKG nuclear translocation [33], [43], [47], the effectors of nuclear EGL-4/PKG activity *in vivo* had been ill-defined. Given the ubiquitous expression of EGL-4, SAEG-1 and SAEG-2, and the requirement of SAEG-1 and SAEG-2 by EGL-4 in multiple physiological processes, our results support a model in which the SAEG-1/SAEG-2 complex mediates EGL-4 activity in the nucleus in diverse tissues. Nevertheless, we noted that loss of *saeg-1* or *saeg-2* function was not equivalent to a complete loss of *egl-4* function (Table 1). This suggests that EGL-4 activity can be mediated by alternative nuclear or cytoplasmic pathways in parallel of SAEG-1 and SAEG-2 (Figure 6). For example, EGL-4 has been reported to modulate gene expression of two chemosensory receptors, *str-1* and *str-3*, through inhibition of the histone deacetylase HDA-4 [48]. It is plausible that EGL-4 engages different histone deacetylase complexes in a context dependent manner.

We took a non-biased genomic approach to identify EGL-4 activity responsive genes. Based on our model, we reasoned that such genes should be repressed upon EGL-4 activation in a SAEG-1/SAEG-2 complex dependent manner. In addition to Y45F10C.2, which regulates egg laying rate, we also identified a group of genes that are involved in lipid metabolism, lipid and sugar transport, and extracellular signaling (Figure 5B; YAH, LCH and HYM, unpublished data). Our results are consistent with the observation that alteration in PKG activity in *Drosophila* also led to changes in lipid and carbohydrate storage and metabolism [49].

How does EGL-4 regulate egg-laying has been an open question since the original isolation of *egl-4* mutants almost 30 years ago [20]. Although the neuronal circuit that controls egg-laying muscles is well-defined [50]–[52], additional mechanisms have been proposed to modulate egg-laying rate in response to environmental stimuli. EGL-4 may be involved in the latter since pharmacological studies failed to assign a role for EGL-4 in the neuronal circuit that controls the egg-laying muscles or at the muscles themselves [20]. Here, we provide experimental evidence that EGL-4 controls egg-laying, at least in part, by modulating the expression of Y45F10C.2, a putative secreted protein from the uterine epithelium. The DUF1505 family, to which Y45F10C.2 belongs, appears to have undergone expansion through gene duplication in *C. elegans*. However, Y45F10C.2 and its duplicate, C08F11.12 are the only family members whose expression is regulated by EGL-4 activity (NXU and HYM, unpublished data). The molecular target of Y45F10C.2 is unknown. It is plausible that Y45F10C.2 can bind to novel cell surface receptors that inhibit egg laying or modulate the function of serotonin, acetylcholine or neuropeptide receptors at the neuromuscular circuit for egg laying.

Our results support a surprising *in vivo* role for the mammalian orthologs of SAEG-1 and SAEG-2. In cell culture systems, TRERF1 has been reported to activate CYP11A1, a gene required for steroidogenesis [41]. TRERF1 also interacts with Dnttip1 and together, they have been implicated in V(D)J recombination by antagonizing the terminal deoxynucleotidyltransferase (TdT) [44]. Another SAEG-1 ortholog, ZNF541, has been implicated in chromatin remodeling during spermatogenesis in mice [40]. While it is hard to reconcile such diverse functions of TRERF1, ZNF541 and Dnttip1, our results implicate that together with HDAC1 or HDAC2, TRERF1 and Dnttip1 may constitute a PKG effector complex in tissues where they are co-expressed, such as the olfactory bulb in mice (Allen Brain Atlas). Notably, EGL-4 is known to be required for chemotaxis and olfactory adaptation in sensory neurons in *C. elegans* [21], [24]. It is plausible that PKG may modulate gene expression in the olfactory bulb via the TRERF1/Dnttip1/HDAC complex upon olfactory stimulation.

While the intracellular cGMP level is tightly regulated by the opposing action of guanylyl cyclase (GC) and phosphodiesterase (PDE) physiologically [1], [2], NO-releasing organic nitrates and PDE inhibitors have been developed to increase cGMP level pharmacologically [1]. This is because the NO-cGMP-PKG pathway is critical for relaxation of smooth muscles and activation of this pathway provides effective therapy for erectile dysfunction, pulmonary hypertension and potentially other diseases associated with abnormal smooth muscle tone. Despite the wide spread use of nitrates and PDE inhibitors, little is known about the effects of sustained elevation of cGMP level and presumably prolonged activation of PKG. Our results suggest that the potential genomic effects of PKG activation should be considered when administering nitrates and PDE inhibitors. Furthermore, the conserved SAEG-1/SAEG-2 histone deacetylase complex represents new molecular targets for the development of chemicals that specifically target cytoplasmic versus nuclear PKG activity.

In summary, our results provide a molecular framework on how nuclear EGL-4/PKG activity triggers wide-spread physiological responses. In *C. elegans*, we expect to identify additional EGL-4 responsive genes that regulate foraging behavior and body length. It is plausible that the SAEG-1/SAEG-2 histone deacetylase complex may engage tissue specific transcription factors, which may be identified through isolation of tissue specific genetic suppressors of the *egl-4(gf)* mutant. Given the deep conservation of EGL-4, SAEG-1 and SAEG-2, our model should be readily applicable in mammals and expand the mode of action of cGMP signaling and its downstream kinase PKG.

## Materials and Methods

### Strains and transgenes

The wild-type strain was Bristol N2. All animals were raised at 20°C. The following alleles and transgenes were used:

LGI: *hda-3(ok1991)*

LGII: *hda-2(ok1479)*

LGIII: *saeg-2(hj9)*, *saeg-2(ok3174)*

LGIV: *egl-4(n479)*, *egl-4(mg410)*, *egl-4(mg410hj32)*, *egl-4(mg410hj40)*, *egl-4(mg410hj33)*

LGV: *saeg-1(hj11)*, *saeg-1(hj12)*, *saeg-1(hj15)*, *saeg-1(hj16)*

LGX: *hda-4(ok518)*

*hjSi15[saeg-2p::saeg-2::GFP-3xFLAG]*Generated by MosSCI, outcrossed 2 times with N2.

*hjIs28[hsp-16-41p::3xFLAG::EGL-4(K162N)::SL2::mCherry::C. briggsae unc-119(+)]*Generated by micro-particle bombardment, outcrossed 2 times with N2.

*hjIs30[hsp-16-41p::3xFLAG::EGL-4(K499A)::SL2::mCherry::C. briggsae unc-119(+)]*Generated by micro-particle bombardment, outcrossed 2 times with N2.

*hjIs46[Y45F10C.2p::Y45F10C.2::SL2::GFP-PEST]*Generated by UV irradiation, outcrossed 5 times with N2.

*hjEx6, hjEx7 [hda-2p::hda-2::SL2::GFP]* (5 ng/µl)*mec-7::rfp* (30 ng/µl) and pBluescript (65 ng/µl) injected into*hda-2(ok1479); egl-4(mg410)*

*hjEx8, hjEx9 [Y45F10C.2p::Y45F10C.2(C36A)::SL2::GFP]* (50 ng/µl)*mec-7::rfp* (30 ng/µl) and pBluescript (20 ng/µl) injected into N2

*hjEx10 [saeg-1p::saeg-1::GFP::saeg-1 3′UTR]*(22.7 kb SalI fragment (coordinates: 10856245–10878915) encompassing the 5′ regulatory region and coding sequence of *saeg-1* from fosmid WRM062aH08 at 5 ng/µl; 8.3 kb ApaI/BamHI fragment (coordinates: 10851160–10858568) from fosmid WRM062aH08 encompassing the 3′ coding sequence and 3′ UTR of *saeg-1* with GFP coding sequence inserted immediately 5′ to the stop codon at 1.8 ng/µl)*mec-7::rfp* (10 ng/µl) and myo-2::mCherry (2.5 ng/µl) injected into

*egl-4(mg410); saeg-1(hj12)*

### Genetic screens

The *egl-4(mg410)* allele was isolated in a previously described genetic screen for mutants that enhanced Nile Red staining of *kat-1(mg368)* mutant animals [35]. Using a SNP-based mapping strategy with the Hawaiian *C. elegans* isolate CB4856 [53], [54], we mapped *mg410* to LGIV between snp_Y38C1BA[2] and snp_pkP4053. Based on annotation of the eight genes in this region, we chose to sequence all exons and exon/intron junction of *egl-4* and identified a single G to T mutation that introduced a K162N (residue numbering as in EGL-4A) substitution in animals carrying the *mg410* allele. This mutation is located in a common exon shared by isoforms a, b, c, and e of *egl-4*.

To isolate genetic suppressors of *egl-4(mg410)*, we mutagenized *egl-4(mg410)* animals with ethyl methane-sulfonate (EMS) by using standard procedures. We screened 34,000 haploid genomes and retrieved both intragenic and extragenic suppressors of *egl-4(mg410)*, which displayed an increase in body length and roaming behavior. Intragenic suppressors are *egl-4* loss-of-function alleles, including *hj32, hj33* and *hj40*. In addition, we isolated extragenic suppressor alleles that fell into two complementation groups. Genetic mapping with the Hawaiian isolate CB4856 placed *saeg-2(hj9)* on LGIII between snp_F54F2[1] and snp_pkP3051. Cosmid rescue and candidate gene sequencing identified a g to a mutation in the splice donor site of the third intron in T23G5.6. Expression of SAEG-2::GFP under the control of *saeg-2* promoter from a single copy transgene (*hjSi15*) also rescued *egl-4(mg410); saeg-2(ok3174)* animals. However, over- expression of SAEG-2::GFP from extra-chromosomal transgenes failed to do so. We mapped *saeg-1(hj11)* to LGV between snp_T21C9[2] and snp_pkP5065. Of the candidate genes we sequenced in this interval, we found mutations in F53H10.2 in *hj11*, *hj12*, *hj15* and *hj16* and we rescued animals carrying *hj12* by injecting the fosmid WRM062aH08, which contained only the coding and regulatory sequences of F53H10.2. We used *hj12* as the reference allele, which introduced a pre-mature stop codon in place of Arg615 (residue numbering as in SAEG-1A) and an 80% decrease in mRNA level as measured by quantitative real-time PCR (YAH and HYM, unpublished data).

### Heat-shock experiments

Heat-shock experiments were performed with synchronized wild-type, *hjIs28* and *hjIs30* young adult animals as judged by vulval morphology. Animals were raised at 20°C on NGM agar plates seeded with OP50 and were heat shocked for 30 mins at 33°C, before returning to 20°C for defined periods of time. 50 animals were picked at each time point for detection of EGL-4 protein by Western blotting.

### Antibodies

Antibodies against EGL-4 were raised in rabbits using a GST-EGL-4A(32-215) fusion protein. EGL-4A(32-215) encompasses the auto-inhibition domain, which is also present in EGL-4 isoforms B, C and E. Antibodies against SAEG-2 were raised in rabbits using full-length SAEG-2 fused to the Pseudomonas Endotoxin at the N-terminus and a poly-histidine tag at the C-terminus. Monoclonal antibodies against the FLAG epitope (clone M2; Sigma) and HA epitope (clone 3F10; Roche) were used for Western blot. For immunoprecipitation, anti-FLAG (clone M2; Sigma) and anti-HA (clone HA-7; Sigma) antibodies conjugated to agarose beads were used.

### Cell and worm lysates preparation and immunoprecipitation

*Drosophila* S2 cells were transfected with copper inducible expression plasmids based on pMT-V5-HisB (Invitrogen) using Effectene Transfection Reagent (Qiagen). 24 hrs after transfection, 700 µM copper sulfate was added to induce gene expression. Cells were harvested 24 hrs later and whole cell lysates were prepared using MAPK buffer (10 mM Tris-HCl pH 8.0, 50 mM NaCl, 1 mM EDTA, 1% NP-40, Roche Complete Protease inhibitors). Human embryonic kidney (HEK293) cells were transfected with expression plasmids based on pcDNA5 (Invitrogen) using Lipofectamine2000 (Invitrogen). Cells were harvested 24 hrs after transfection and whole cell lysates were prepared using MAPK buffer. Immunoprecipitations with agarose beads conjugated with anti-FLAG or anti-HA antibodies were carried out at 4°C for 2.5 hrs.

Mixed-stage worms grown in liquid culture with *E. coli* HB101 were used for preparation of worm lysates and immunoprecipitation as described [55].

### *In vitro* kinase assay

Autophosphorylation of EGL-4 was monitored using FLAG-tagged EGL-4 that was expressed in *Drosophila* S2 cells. After immunoprecipitation, anti-FLAG agarose beads were washed 4 times with MAPK buffer and once with kinase buffer (10 mM HEPES pH 7.4, 5 mM MgCl_2_, 1 mM DTT, Halt phosphatase inhibitors cocktail (Pierce)). *In vitro* phosphorylation was allowed to proceed in the presence of 1 µCi ^32^P ATP in kinase buffer at 30°C for 20 mins. Agarose beads were washed 4 times with kinase buffer and bound proteins were released by boiling in LDS sample buffer (Invitrogen), separated by SDS-PAGE and visualized by autoradiography.

### Measurement of foraging behavior

For automated worm tracking using the Worm Tracker program [37], 5 1-day old adult animals were monitored at room temperature in a 15-min period in each trial. Animals were allowed to move freely on a 6 cm NGM plate freshly seeded with *E. coli* OP50 on the entire surface. Movies were taken on a Lumar dissecting microscope (Zeiss) equipped with a CCD camera with a field of view of 2.2 cm×1.7 cm. Animals that moved out of the field were defined as new objects by the Worm Tracker program. We assigned an animal as dwelling if it exhibited angular velocity ≥110°/s or ≤ –110°/s, for a duration ≥30 s. We assigned an animal as roaming if it was not engaged in dwelling as described above.

Foraging behavior over 18 hrs was measured as the area of bacterial lawn that was explored by a single worm on 6 cm NGM plates seeded with *E. coli* OP50 (∼2 cm diameter) at 20°C. An image of the bacterial lawn was taken on a Lumar dissecting microscope (Zeiss) equipped with a CCD camera and the area covered by worm tracks was measured using Axiovision (Zeiss).

### MudPIT analysis

TCA-precipitated proteins were urea-denatured, reduced, alkylated and digested with endoproteinase Lys-C (Roche) followed by modified trypsin (Roche) as described [45]. Fully automated 10-step MudPIT runs were carried out on a linear ion trap mass spectrometer (ThermoFinnigan) equipped with a nano-LC electrospray ionization source. Tandem mass (MS/MS) spectra were interpreted using SEQUEST [56] against a database combining 30552 non-redundant human proteins (NCBI, 2008-03-04 release), 162 usual contaminants, and both epitope-tagged mouse Dnttip and ZNF541, as well as 30714 randomized amino acid sequences to estimate false discovery rates (FDRs). Peptide/spectrum matches were sorted and selected using DTASelect [57]. FDRs at the protein and peptide levels were both less than 1%. To estimate relative protein levels, Normalized Spectral Abundance Factors (dNSAFs) were calculated for each detected protein as described [58].

### Gene expression profiling

Three independent populations of each strain were harvested at late L4/young adult stage. Total RNA was extracted using TRI reagent (Molecular Research Center) according to manufacturer's instructions. Microarray analysis was performed using Affymetrix GeneChip *C. elegans* Genome Arrays. Biotinylated cRNA was prepared from 300 ng Total RNA using the MessageAmp III kit according to the manufacturer instructions (Ambion). Data was analyzed using the R statistical environment. CEL files resulting from array analysis were interpreted and normalized using RMA [59]. The linear modeling package Limma [60] was used to determine significant gene expression differences based on a moderated t-statistic.

### Real-time PCR

At least two independent populations of each strain were harvested at late L4/young adult stage. Total RNA was extracted using TRI reagent (Molecular Research Center) according to manufacturer's instructions. Genomic DNA contamination was removed using the TURBO DNA-free kit (Ambion) and reverse transcribed using the RETROscript kit (Ambion). The cDNA was subjected to real time PCR analysis using the IQ SYBR Green supermix (Bio-Rad) on an iCycler (Bio-Rad). Each cDNA sample was amplified in triplicate reactions. The primers for Y45F10C.2 and the internal control rpl-32 were checked for specificity by direct sequencing of the PCR products and tested for efficiency with a dilution series of the template. All values were normalized against the *rpl-32* gene whose expression does not vary under our experimental conditions. Fold change was calculated using the Pfaffl method.

## Supporting Information

Figure S1 (A) Expression level of EGL-4 in lysates prepared from strains of indicated genotypes using anti-EGL-4 antibody. The antibody also recognized a non-specific protein (marked by an asterisk). The α-tubulin blot served as loading control. (B) Expression level of EGL-4 in lysates prepared from transgenic animals carrying *hjIs30[hsp::3xFLAG::EGL-4(K499A)::SL2::mCherry]* at specified time after heat shock at 33^∘^C for 30 mins. Endogenous and FLAG-tagged EGL-4 protein was detected using anti-EGL-4 antibody. The α-tubulin blot served as loading control. (C) Expression level of EGL-4 in lysates prepared from transgenic animals carrying *hjIs28[hsp::3xFLAG::EGL-4(K162N)::SL2::mCherry]* at specified time after heat shock at 33^∘^C for 30 mins. Endogenous and FLAG-tagged EGL-4 protein was detected using anti-EGL-4 antibody. The α-tubulin blot served as loading control. (D) Foraging behavior of wild-type, *hjIs28* and *saeg-2(ok3174); hjIs28* animals that were not heat-shocked (nHS), 2 hrs after heatshock (HS-2hrs) or 24 hrs after heatshock (HS-24hrs). Quantitation of behavior was performed as in Figure 1A. 5 animals were included in each trial. Total number of trials: n = 5 for each treatment of each strain except *saeg-2;hjIs28* nHS (n = 6). (Mean+SD; *, *p<0.05* t-test). (E) Number of eggs retained in uterus in wild-type, *hjIs28* and *saeg-2(ok3174); hjIs28* animals that were not heat-shocked (nHS), 2 hrs after heatshock (HS-2hrs) or 24 hrs after heatshock (HS-24hrs). Total number of animals for each treatment of each strain: n = 20 (Mean+SD; *, *p<0.05* t-test).
(PDF)

Figure S2 (A) The *egl-4* mRNA level in strains of indicated genotypes, measured by real-time PCR. Results shown are derived from two independent mRNA samples for each strain assayed in triplicates. The mRNA level in wild-type (WT) animals was set as 1. (B) Expression level of EGL-4 in lysates prepared from strains of indicated genotypes using anti-EGL-4 antibody. The antibody also recognized a non-specific protein (marked by an asterisk). The α-tubulin blot served as loading control. (C) Expression level of SAEG-2 in lysates prepared from strains of indicated genotypes using anti-SAEG-2 antibody. The α-tubulin blot served as loading control. (D) SAEG-2::GFP nuclear localization is not affected by EGL-4 activity. *hjSi15[saeg-2p::saeg-2::GFP-3xFLAG]* single copy transgene was introduced into *saeg-2(ok3174)*, *egl-4(mg410); saeg-2(ok3174)* and *egl-4(n479); saeg-2(ok3174)* mutant backgrounds. Confocal images of the head region centering on neurons at the nerve ring (marked by brackets) are shown.
(PDF)

Figure S3 Immunostaining of dissected intestine of 1-day old adult wild type, *egl-4(mg410)* and *egl-4(n479)* animals using anti-EGL-4 antibody. Scale bar  =  10 mm.
(PDF)

Figure S4 (A) Co-immunoprecipitation of FLAG-tagged SAEG-2 (FLAG-SAEG-2) but not FLAG-tagged yellow fluorescent protein Venus (FLAG-Venus) with HA-tagged SAEG-2 (HA-SAEG-2) upon co-expression in *Drosophila* S2 cells. (B) Co-immunoprecipitation of FLAG-tagged EGL-4 (FLAG-EGL-4) but not FLAG-tagged Venus (FLAG-Venus) with HA-tagged SAEG-2 (HA-SAEG-2) upon co-expression in *Drosophila* S2 cells. In the same experiment, co-immunoprecipitation of HA-tagged FLAG-EGL-4 with SAEG-1 (HA-SAEG-1) was reproduced. (C) Co-immunoprecipitation of FLAG-tagged PKG-Iβ(S64D) (FLAG-PKG) but not FLAG-tagged Venus (FLAG-Venus) with HA-tagged ZNF541 (HA-ZNF541) upon co-expression in HEK293 cells. (D) Co-immunoprecipitation of FLAG-tagged PKG-Iβ(S64D) (FLAG-PKG) and FLAG-tagged ZNF541 (FLAG-ZNF541) but not FLAG-tagged Venus (FLAG-Venus) with HA-tagged Dnttip1 (HA-Dnttip1) upon co-expression in HEK293 cells.
(PDF)

Figure S5 Immunostaining of dissected uteri of 1-day old adult wild type and *egl-4(n479)* animals using anti-EGL-4 antibodies. (A–B) Nuclear staining was detected with anti-EGL-4 antibodies in a wild-type uterine epithelial cell (red arrowhead). (C–D) Nuclear staining was absent in an *egl-4(n479)* uterine epithelial cell (red arrowhead), demonstrating the specificity of the antibodies. Similar staining pattern was observed in at least 3 other samples for each genotype. Note background cytoplasmic staining in somatic gonadal cells in (A) and (C) but specific nuclear staining of the same cells in (A) (white arrowheads). Boxed areas in (A) and (C) were imaged at higher magnification and shown in (B) and (D). Scale bar  =  10 mm.
(PDF)
