# Supplementary figures and images for: Nuclear cGMP-Dependent Kinase Regulates Gene Expression via Activity-Dependent Recruitment of a Conserved Histone Deacetylase Complex
Source: PLoS Genet. 2011 May 5;7(5):e1002065. doi: 10.1371/journal.pgen.1002065 (PMC3088716; doi:10.1371/journal.pgen.1002065)

Figure S1 Hao et al

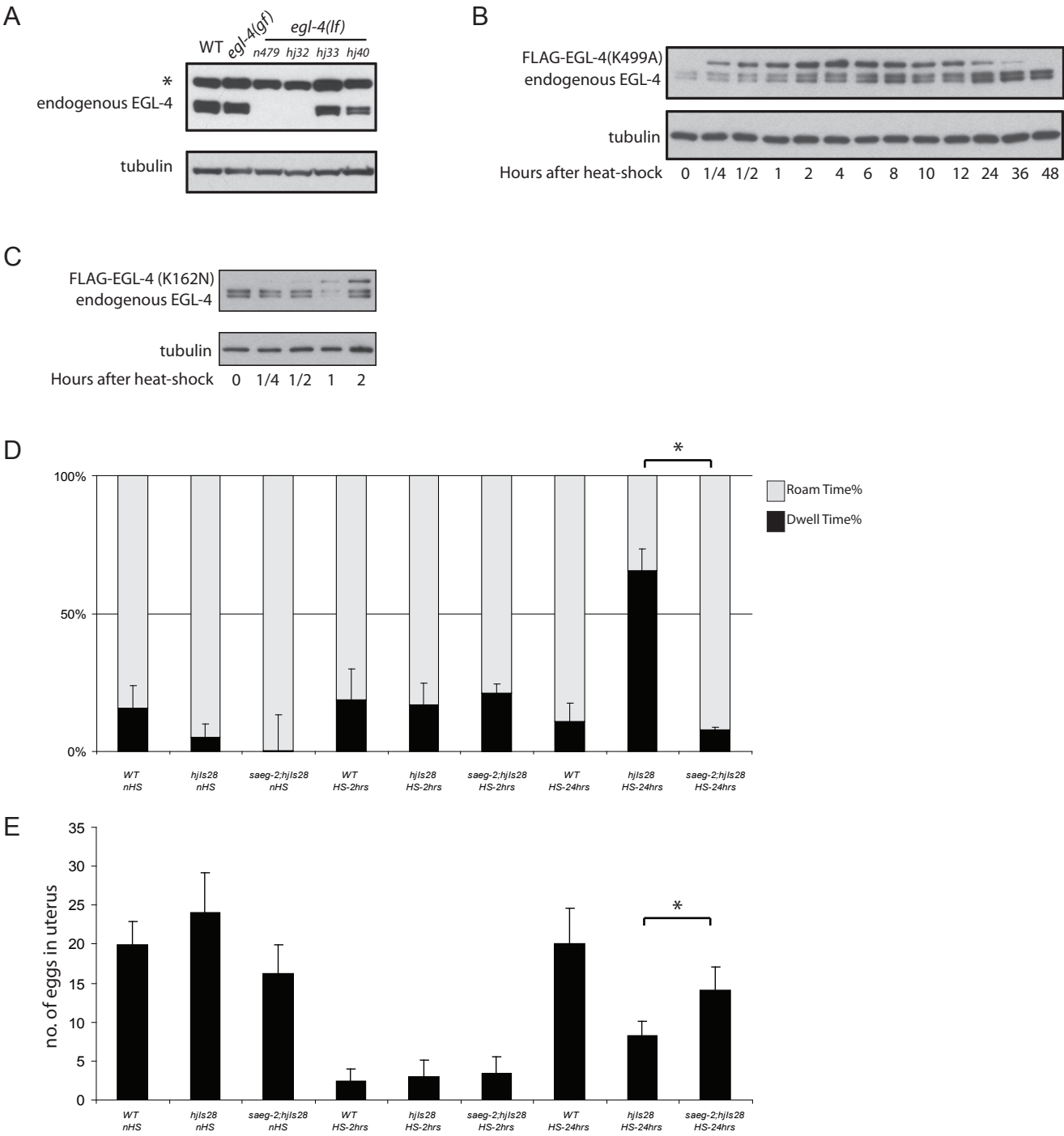

Supplement: Figure S1 — (A) Expression level of EGL-4 in lysates prepared from strains of indicated genotypes using anti-EGL-4 antibody. The antibody also recognized a non-specific protein (marked by an asterisk). The α-tubulin blot served as loading control. (B) Expression level of EGL-4 in lysates prepared from transgenic animals carrying hjIs30[hsp::3xFLAG::EGL-4(K499A)::SL2::mCherry] at specified time after heat shock at 33∘C for 30 mins. Endogenous and FLAG-tagged EGL-4 protein was detected using anti-EGL-4 antibody. The α-tubulin blot served as loading control. (C) Expression level of EGL-4 in lysates prepared from transgenic animals carrying hjIs28[hsp::3xFLAG::EGL-4(K162N)::SL2::mCherry] at specified time after heat shock at 33∘C for 30 mins. Endogenous and FLAG-tagged EGL-4 protein was detected using anti-EGL-4 antibody. The α-tubulin blot served as loading control. (D) Foraging behavior of wild-type, hjIs28 and saeg-2(ok3174); hjIs28 animals that were not heat-shocked (nHS), 2 hrs after heatshock (HS-2hrs) or 24 hrs after heatshock (HS-24hrs). Quantitation of behavior was performed as in Figure 1A. 5 animals were included in each trial. Total number of trials: n = 5 for each treatment of each strain except saeg-2;hjIs28 nHS (n = 6). (Mean+SD; *, p<0.05 t-test). (E) Number of eggs retained in uterus in wild-type, hjIs28 and saeg-2(ok3174); hjIs28 animals that were not heat-shocked (nHS), 2 hrs after heatshock (HS-2hrs) or 24 hrs after heatshock (HS-24hrs). Total number of animals for each treatment of each strain: n = 20 (Mean+SD; *, p<0.05 t-test). (PDF) [file pgen.1002065.s001.pdf]

Figure S2    Hao *et al*

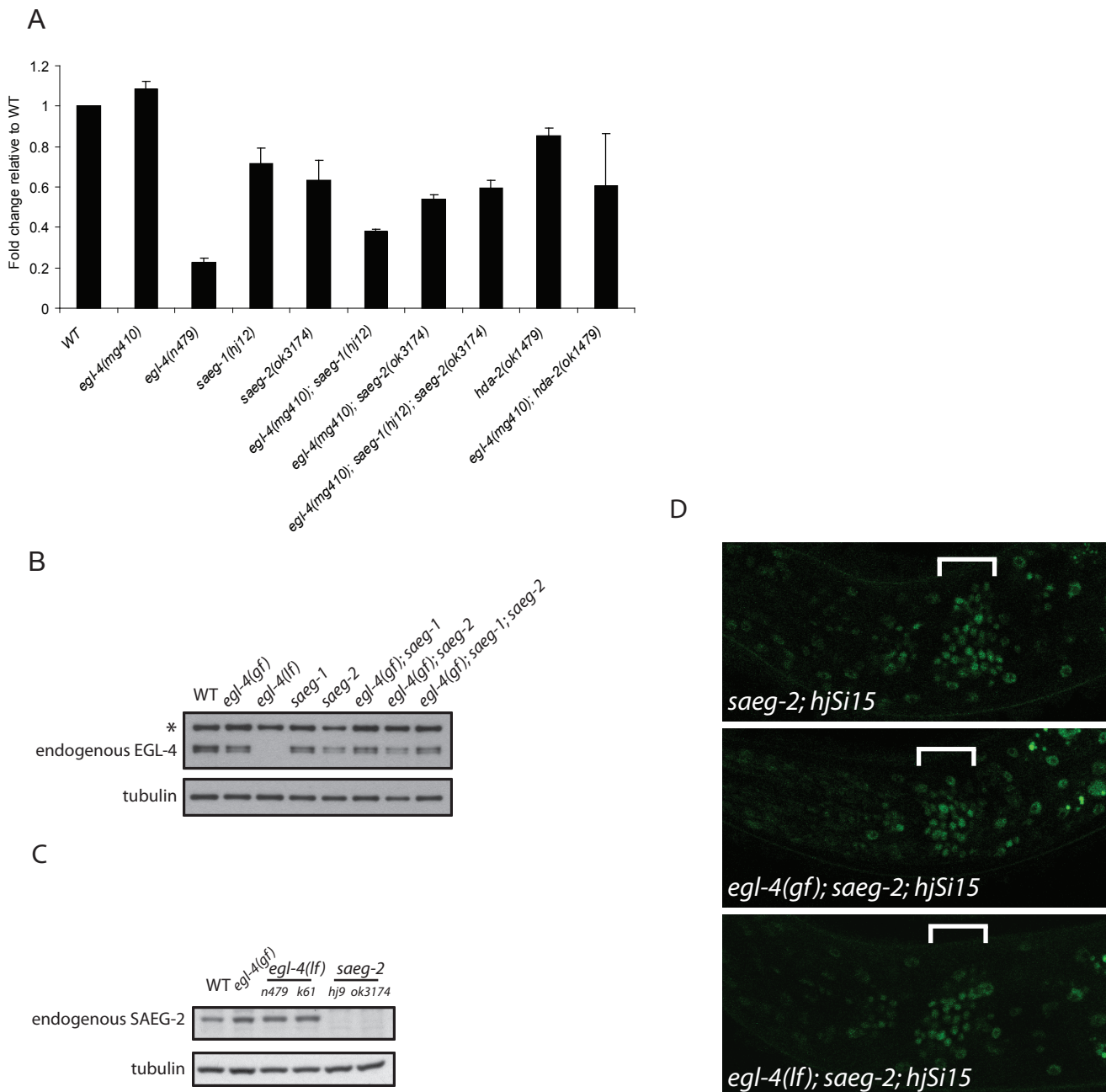

Supplement: Figure S2 — (A) The egl-4 mRNA level in strains of indicated genotypes, measured by real-time PCR. Results shown are derived from two independent mRNA samples for each strain assayed in triplicates. The mRNA level in wild-type (WT) animals was set as 1. (B) Expression level of EGL-4 in lysates prepared from strains of indicated genotypes using anti-EGL-4 antibody. The antibody also recognized a non-specific protein (marked by an asterisk). The α-tubulin blot served as loading control. (C) Expression level of SAEG-2 in lysates prepared from strains of indicated genotypes using anti-SAEG-2 antibody. The α-tubulin blot served as loading control. (D) SAEG-2::GFP nuclear localization is not affected by EGL-4 activity. hjSi15[saeg-2p::saeg-2::GFP-3xFLAG] single copy transgene was introduced into saeg-2(ok3174), egl-4(mg410); saeg-2(ok3174) and egl-4(n479); saeg-2(ok3174) mutant backgrounds. Confocal images of the head region centering on neurons at the nerve ring (marked by brackets) are shown. (PDF) [file pgen.1002065.s002.pdf]

Figure S3     Hao *et al*

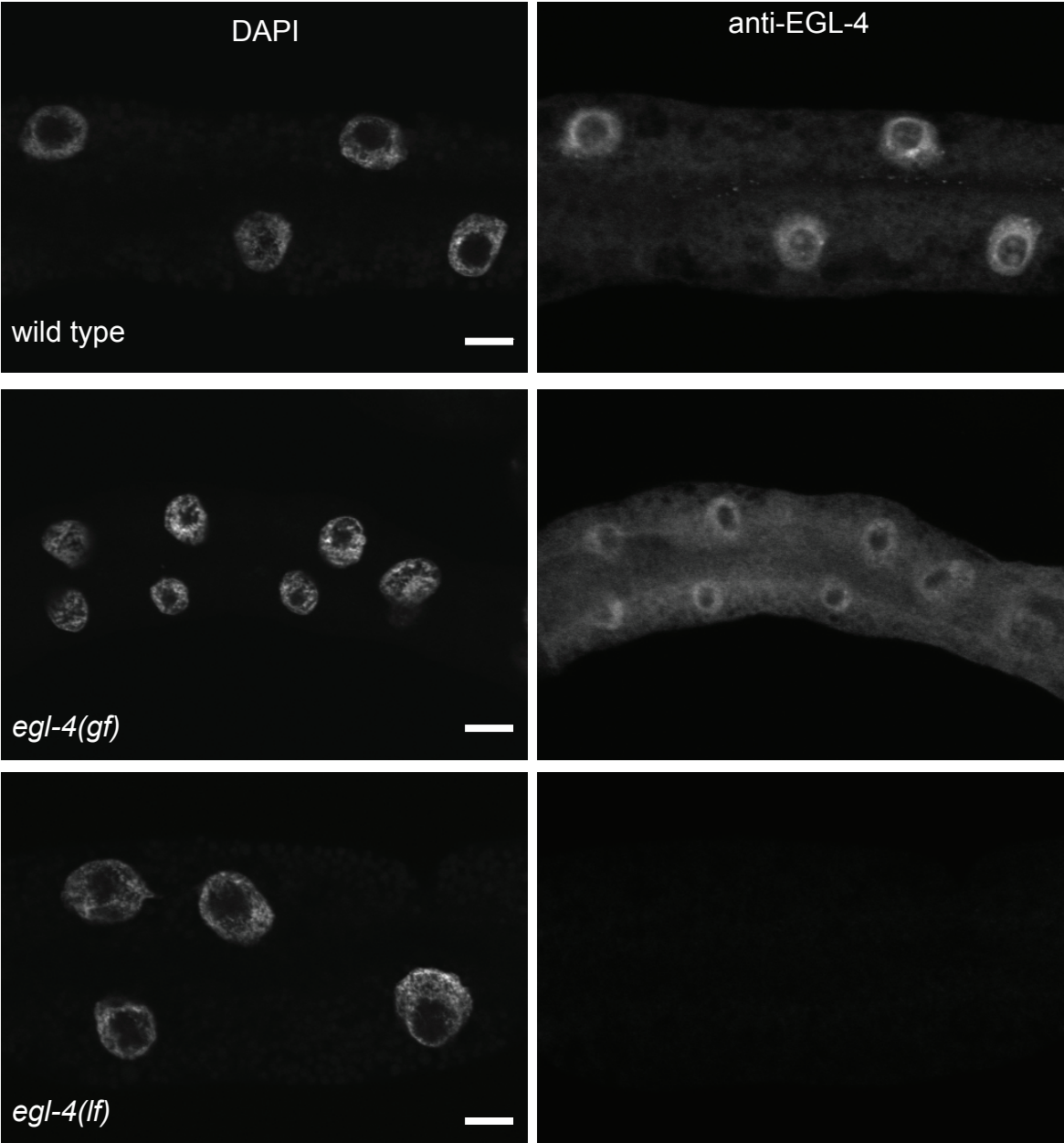

Supplement: Figure S3 — Immunostaining of dissected intestine of 1-day old adult wild type, egl-4(mg410) and egl-4(n479) animals using anti-EGL-4 antibody. Scale bar = 10 mm. (PDF) [file pgen.1002065.s003.pdf]

Figure S4     Hao *et al*

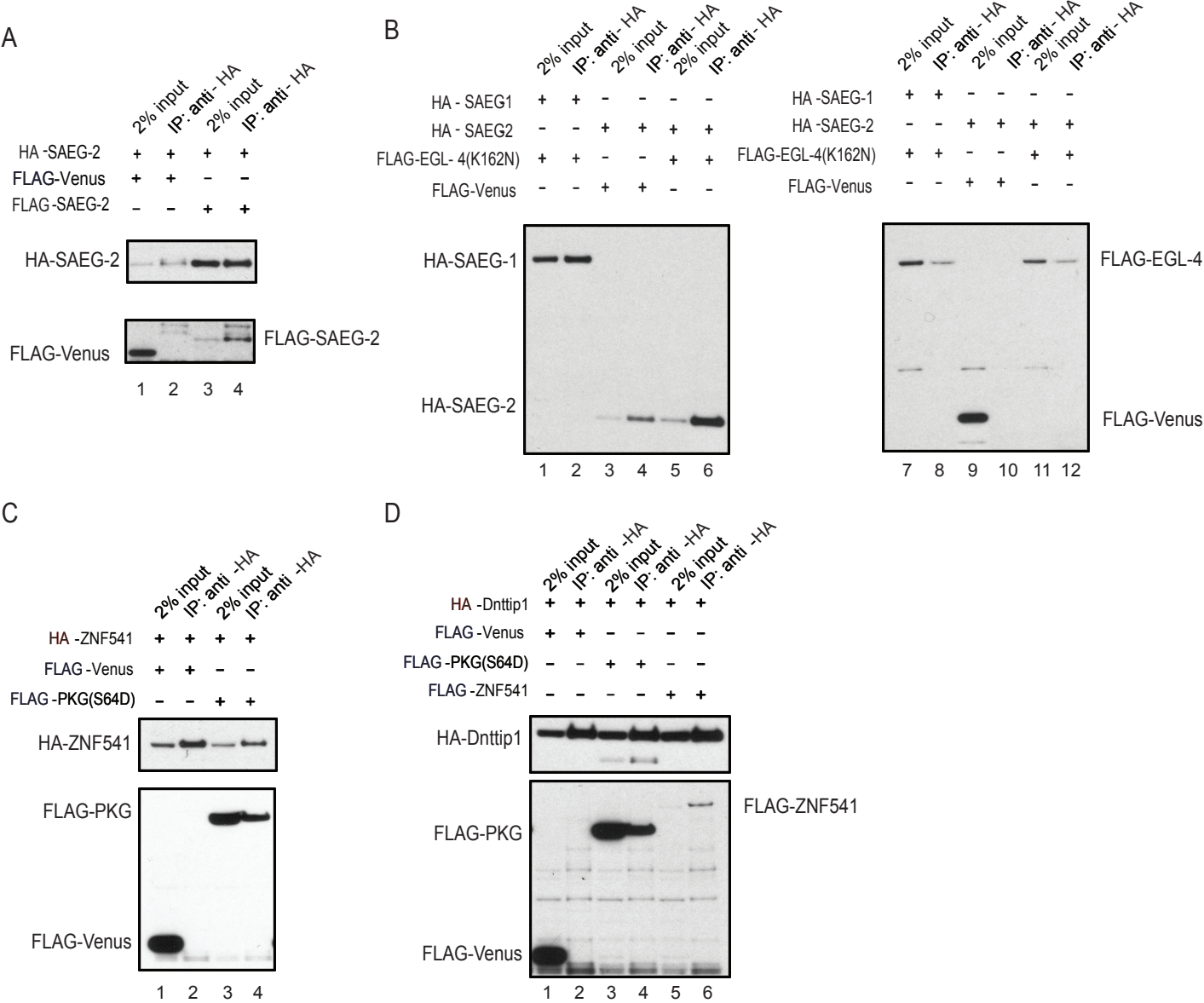

Supplement: Figure S4 — (A) Co-immunoprecipitation of FLAG-tagged SAEG-2 (FLAG-SAEG-2) but not FLAG-tagged yellow fluorescent protein Venus (FLAG-Venus) with HA-tagged SAEG-2 (HA-SAEG-2) upon co-expression in Drosophila S2 cells. (B) Co-immunoprecipitation of FLAG-tagged EGL-4 (FLAG-EGL-4) but not FLAG-tagged Venus (FLAG-Venus) with HA-tagged SAEG-2 (HA-SAEG-2) upon co-expression in Drosophila S2 cells. In the same experiment, co-immunoprecipitation of HA-tagged FLAG-EGL-4 with SAEG-1 (HA-SAEG-1) was reproduced. (C) Co-immunoprecipitation of FLAG-tagged PKG-Iβ(S64D) (FLAG-PKG) but not FLAG-tagged Venus (FLAG-Venus) with HA-tagged ZNF541 (HA-ZNF541) upon co-expression in HEK293 cells. (D) Co-immunoprecipitation of FLAG-tagged PKG-Iβ(S64D) (FLAG-PKG) and FLAG-tagged ZNF541 (FLAG-ZNF541) but not FLAG-tagged Venus (FLAG-Venus) with HA-tagged Dnttip1 (HA-Dnttip1) upon co-expression in HEK293 cells. (PDF) [file pgen.1002065.s004.pdf]

Figure S5    Hao *et al*

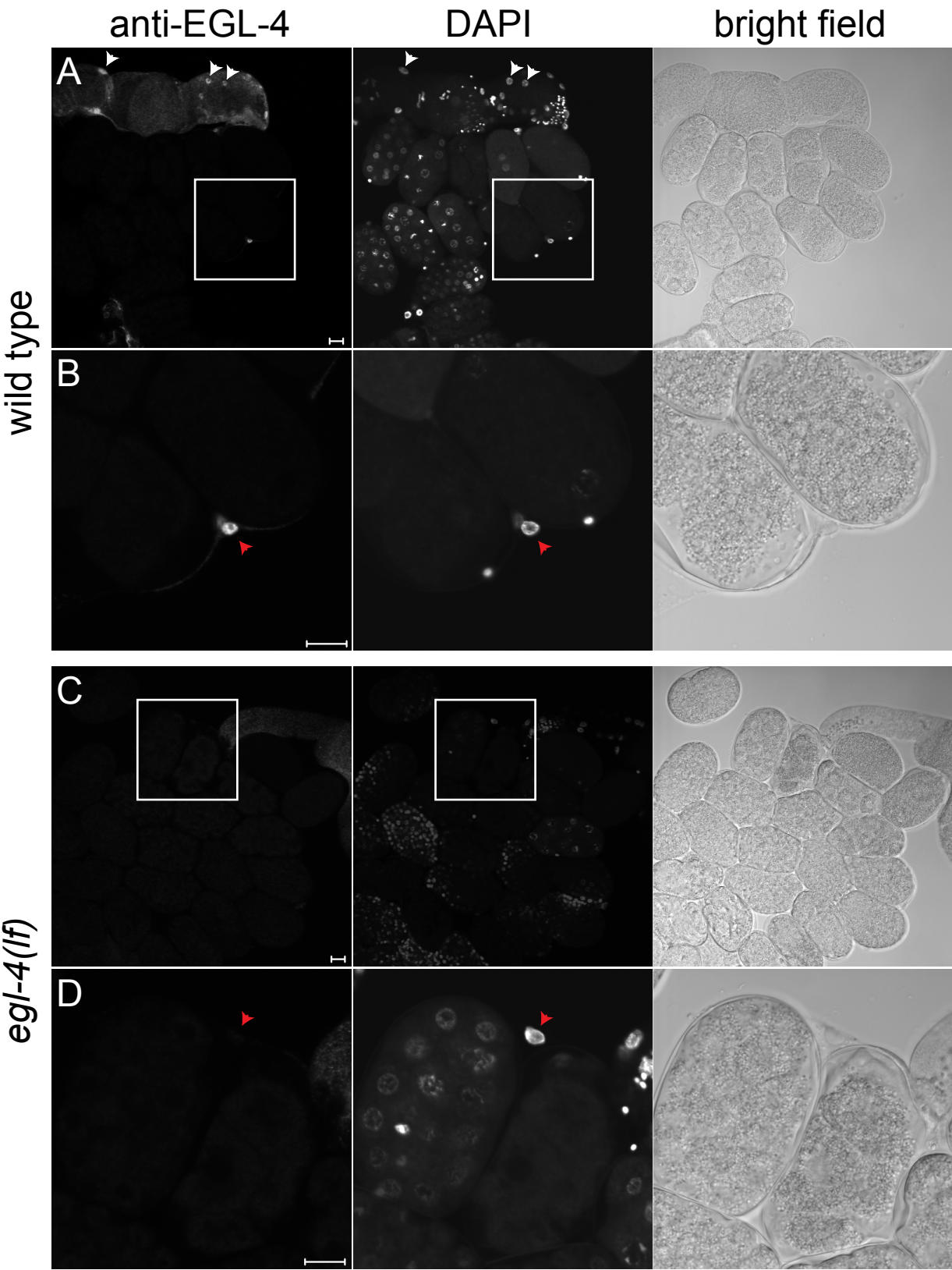

Supplement: Figure S5 — Immunostaining of dissected uteri of 1-day old adult wild type and egl-4(n479) animals using anti-EGL-4 antibodies. (A–B) Nuclear staining was detected with anti-EGL-4 antibodies in a wild-type uterine epithelial cell (red arrowhead). (C–D) Nuclear staining was absent in an egl-4(n479) uterine epithelial cell (red arrowhead), demonstrating the specificity of the antibodies. Similar staining pattern was observed in at least 3 other samples for each genotype. Note background cytoplasmic staining in somatic gonadal cells in (A) and (C) but specific nuclear staining of the same cells in (A) (white arrowheads). Boxed areas in (A) and (C) were imaged at higher magnification and shown in (B) and (D). Scale bar = 10 mm. (PDF) [file pgen.1002065.s005.pdf]
